# Evaluating The Economic Impact Of Marriage Versus Cohabitation In Same-Sex Couples Age 50+

**DOI:** 10.1093/geroni/igab046.3172

**Published:** 2021-12-17

**Authors:** Setarreh Massihzadegan, Jan Mutchler

**Affiliations:** University of Massachusetts Boston, Boston, Massachusetts, United States

## Abstract

Utilizing the first set of 5-year American Community Survey data available since the United States’ legalization of same-sex marriage in mid-2015, this poster investigates the economic security of older adults (age 50+) in same-sex marriages compared to those in same-sex partnerships who are cohabiting but not married. Viewed through the lens of cumulative disadvantage theory, we consider differences in the economic circumstances of same-sex couples by gender and by geographic location. Findings point to gender differences in economic well-being, but relatively few differences based on marital status. For example, rates of low income are somewhat higher among female couples than among their male counterparts, but marital status differences are not substantial. These findings suggest that the benefits of being married that have long been recognized among older adults may not extend equally to same-sex couples. Findings are discussed with respect to the emerging salience of marriage within the LGBTQ older community, future research opportunities, and important policy implications.

